# Towards accurate genomic detection of fungal antimicrobial resistance: progress in fungal resistance databases and bioinformatic tools

**DOI:** 10.1099/mgen.0.001710

**Published:** 2026-05-20

**Authors:** Andrew P Gador-Whyte, Johanna Rhodes, Rhys A Farrer, Sibbe L Bakker, Wytamma Wirth, Rosa C Coldbeck-Shackley, Benjamin P Howden, Jason C Kwong, Norelle L. Sherry, Torsten Seemann

**Affiliations:** 1 Microbiological Diagnostic Unit Public Health Laboratory, Department of Microbiology & Immunology, University of Melbourne at the Peter Doherty Institute for Infection and Immunity, Melbourne, Australia; 2 Department of Infectious Diseases and Immunology, Austin Health, Heidelberg, Australia; 3 Department of Microbiology and Immunology, University of Melbourne, at the Peter Doherty Institute for Infection and Immunity, Melbourne, Australia; 4 School of Biosciences, University of Birmingham, Birmingham, UK; 5 MRC Centre for Medical Mycology, University of Exeter, Exeter, UK; 6 NIHR Exeter Biomedical Research Centre, University of Exeter, Exeter, UK; 7 Department of Theoretical Biology and Bioinformatics, Utrecht University, Utrecht, Netherlands; 8 Centre for Pathogen Genomics, University of Melbourne, Parkville, Australia; 9 Microbiology and Infectious Diseases, South Australia Pathology, Adelaide, Australia; 10 Department of Microbiology, Royal Melbourne Hospital, Parkville, Australia; 11 Department of Infectious Diseases, University of Melbourne, at the Peter Doherty Institute for Infection and Immunity, Melbourne, Australia

**Keywords:** databases as topic, drug resistance, fungal, genetics, genomics, workflow

## Abstract

Fungal antimicrobial resistance (fAMR) is increasing worldwide and is recognized as a global health priority by the World Health Organization. The emergence of *Candidozyma (Candida) auris* and other resistant fungal pathogens presents a risk to critically ill patients. Whole-genome sequencing has the potential to improve public health and clinical surveillance for fAMR and could enable more rapid detection. In this review, we discuss the mechanisms of fAMR and the strengths and limitations of the currently available databases and bioinformatic tools for the detection of fAMR from genomic data. We identify current gaps, preferred characteristics of genomic fAMR databases and tools and future directions for development to enable validated fAMR prediction in the public health context.

**Impact Statement**

Fungal antimicrobial resistance (fAMR) is increasing, but our understanding of fungal genomics has lagged behind that of bacteria and viruses. The emergence of *Candidozyma auris* and other fungal pathogens harbouring fAMR has highlighted the urgency of public health surveillance for, and more rapid detection of, fAMR. This review considers why the implementation of genomic fAMR detection in public health and clinical microbiology has been challenging to date. We provide an update on important recent developments in this field. We highlight an important gap in the current genomic fAMR field, the lack of validated workflows for implementation of genomic fAMR detection. This review outlines a pathway to implementation and highlights those areas needing development to enable prospective genomic surveillance and ultimately direct detection of fAMR.

## Data Summary

All supporting data have been provided within the article. All data are available in the cited literature.

## Introduction

Fungal antimicrobial resistance (fAMR) is a global health priority recognized by the World Health Organization (WHO) in their 2022 Priority Fungal Pathogens List [1]. The past decade has witnessed the global emergence of *Candidozyma (Candida) auris* as a multidrug-resistant, healthcare-associated pathogen [2]. Triazole resistance in *Aspergillus fumigatus* acquired from the environment is a particular concern for immunocompromised patients in many countries and represents a One Health challenge [3,4]. In addition, a terbinafine-resistant clone of *Trichophyton indotineae* as a cause of severe dermatophytosis has been recognized, also highlighting the importance of antifungal stewardship [5]. Increasing triazole resistance in common pathogens including *Candida parapsilosis*, *Nakaseomyces glabratus* (*Candida glabrata*) and *Candida tropicalis* is of substantial concern, especially for the treatment of healthcare-associated infections [6,8].

Traditional, culture-based techniques for detecting fAMR are slow and relatively insensitive [9]. Phenotypic antifungal susceptibility testing (AFST) also requires specialized skills, particularly for moulds, and can be affected by inter-operator and inter-laboratory variation [10]. Whole-genome sequencing (WGS) is a powerful technology that could improve surveillance for antifungal resistance, provide a greater understanding of how resistant fungal pathogens spread and enable culture-independent diagnosis for clinical use and potentially rapid fAMR detection [11,13]. Genomic detection of antimicrobial resistance (AMR) in bacterial pathogens is well-established in public health microbiology [14] and emerging in clinical microbiology [15], but the field of genomic antifungal resistance detection is less mature: fungal pathogens are relatively under-researched, there is a paucity of WGS data, no standardized genomic workflows and genomic fAMR mechanisms can be complex [16].

Identification of AMR from genomic data requires (i) databases of genomic determinants (such as mutations and genes) associated with phenotypic susceptibility data and (ii) bioinformatic software capable of identifying these variants from the genome sequence data [17]. To date, antifungal resistance databases have been limited in both breadth and depth, and few software tools have been developed to identify antifungal resistance determinants from fungal genomes. This is changing, however, with the recent publication of large curated genomic antifungal resistance databases and fungal variant calling and resistance reporting workflows. This review compares the databases and tools currently available to identify the current gaps and future directions for development, with a view ultimately to integrating genomic antifungal resistance detection into validated workflows in clinical and public health microbiology.

## Fungi and invasive fungal infections

The kingdom Fungi is enormously diverse and includes many organisms with essential roles in nature as organic matter decomposers [18]. Several hundred fungal species are potentially pathogenic to humans, although most serious human infections are due to a small number of taxa, particularly *Aspergillus*, and yeast genera including *Candida*, *Cryptococcus* and *Pneumocystis* [19]. Clinical fungal infections range in severity from superficial skin and mucous membrane infections (such as dermatophytosis) to life-threatening invasive pulmonary infections (e.g. *A. fumigatus*), bloodstream infections (e.g. *C. albicans*) and disseminated mould disease (e.g. *Lomentospora prolificans*) [20,21]. Patients requiring intensive care unit admission, cancer treatment and organ transplantation are at greatest risk [21]. Transmission of resistant fungi, particularly *C. auris* and *C. parapsilosis*, threatens the effectiveness of empiric antifungal treatment [2,22].

Surveillance for emergence of fAMR, identifying its sources and detecting transmission of antifungal-resistant pathogens in healthcare, the community and the environment are critical priorities to protect the community and vulnerable patient cohorts from difficult-to-treat fungal infections [23].

Additionally, given the high mortality associated with invasive fungal infections, early commencement of active antifungal treatment can be life-saving [24]. As a result, early diagnosis and early detection of fAMR are crucial for individual patient treatment.

## Antifungal agents: targets, drugs, mechanisms of resistance and current susceptibility testing methods

Fungi, like humans, are eukaryotes, and the similarity of fungal and human cells means there are a limited number of available targets for antifungal drugs that do not impact similar cellular functions in human cells [25]. Fungal cells do, however, have unique features which can be targeted by antifungal medications. Fungal cell membranes contain ergosterol (in contrast to cholesterol in human cell membranes) [26] which is the target of the triazole, allylamine and polyene antifungal drug classes [27]. The rigid fungal cell wall incorporates the carbohydrate 1,3-*β*-d-glucan (targeted by the echinocandins and the new triterpenoid class) and mannoproteins cross-linked to glucans (this cross-linking is the target of the novel agent fosmanogepix) [27,28]. Differences in nucleotide metabolism in fungal cells also enable the pyrimidine analogue flucytosine to be selectively toxic to fungi [29].

**Fig. 1.**
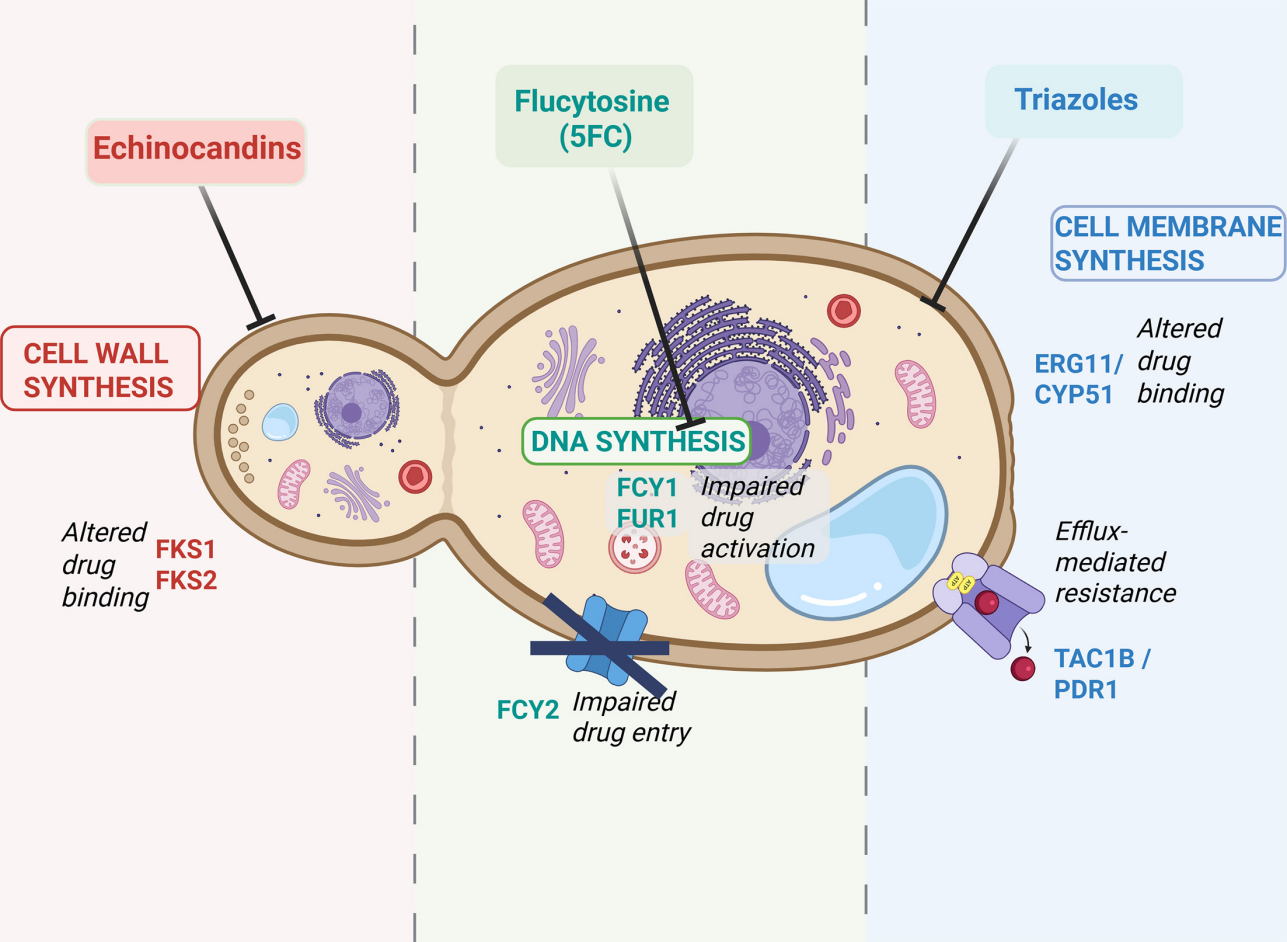
Simplified diagram of the fungal cell, antifungal drugs targets, key genes and broad groups of resistance mechanisms. Created in BioRender. Gador-whyte, A. (2025) https://BioRender.com/g2tt68g

Because of their clinical importance and the increasing rates of resistance, the triazoles and the echinocandins have been the major focus of antifungal resistance research. Examples of each antifungal class and common mechanisms of resistance are summarized in Fig. 1 [9,27, 29,34] and Table 1. For the echinocandins (Fig. 1, left panel), the most important resistance mechanism is drug binding site modifications in the 1,3-*β*-d-glucan synthase enzymes, resulting from point mutations in the genes *FKS1* and *FKS2* [35,36]. For flucytosine (Fig. 1, central panel), loss-of-function mutations in *FCY2* lead to impaired drug entry, whilst mutations in *FCY1* and *FUR1* reduce drug activation and so its ability to interrupt nucleic acid synthesis [37]. For triazoles (Fig. 1, right panel), mechanisms include drug binding site modifications in the lanosterol 14*α*-demethylase gene (e.g. *ERG11* in *C. albicans*) due to point mutations leading to accumulation of a fungistatic compound [38], overexpression of *ERG11* due to copy number variation or *UPC2* gain-of-function mutations [38,39] and enhanced efflux due to gain-of-function mutations in transcription factors (e.g. *PDR1* in *N. glabratus* and *TAC1B* in *Candidozyma auris*) [36,40]. Not shown in Fig. 1 are mechanisms of polyene resistance, which are complex and poorly understood but may include point mutations in ergosterol synthesis pathway genes such as *ERG3*, *ERG6* or *ERG11* [35,40] and allylamine resistance, usually due to *ERG1 (SQLE*) point mutations [41].

**Table 1.** Summary of key antifungal classes and mechanisms of resistance [27,29,34, 75]

Antifungal class	Example	Site of action	Specific mechanism of action	Example of resistance mechanism	Example gene	Example species/genus	Typical genomic modification types
Triazole	Fluconazole Voriconazole Posaconazole	Cell membrane	Inhibition of lanosterol 14*α*-demethylase with fungistatic metabolite accumulation	Target modification	*ERG11* *CYP51A*	*Candida* spp. *A. fumigatus*	SNP SNP+promoter tandem repeat
				Target overexpression	*ERG11* *UPC2*	*Candida* spp. “	Copy number variation Aneuploidy/isochromosome Transcription factor mutation causing *ERG11* overexpression
				Drug efflux	*TAC1B* *CDR1*	*Candida* spp. “	SNP (transcription factor) Overexpression (efflux pump gene)
Echinocandin	Anidulafungin	Cell wall	Inhibition of glucan synthase	Target enzyme mutation	*FKS1* *FKS2*	*Candida* spp. *N. glabratus*	SNP
Polyene	Amphotericin B Nystatin	Cell membrane	Binding to or extracting ergosterol from cell membrane	Reduced expression Loss of function	*ERG11* *ERG3* *ERG6*	*C. auris* “ “	Various Loss-of-function mutations
Pyrimidine analogue	Flucytosine	DNA synthesis	DNA chain termination	Impaired prodrug activation Impaired drug incorporation Reduced drug entry	*FCY1* *FUR1* *FCY2*	*Candida* spp. *N. glabratus* *N. glabratus*	SNP SNP SNP
Allylamines	Terbinafine	Cell wall	Inhibition of squalene epoxidase	Target enzyme mutation	*SQLE (ERG1*)	*T. indotineae*	SNP

For a fuller treatment of phenotypic AFST methods, see the reviews by Berkow *et al.* [42] and Durand *et al.* [10]. In brief, current AFST methods performed for invasive fungal infections are predominantly culture-based. Culture isolation is relatively insensitive for fungal pathogens [43,45], and culture-based AFST tends to have a prolonged turnaround time as a result of slow fungal growth [10,12]. AFST is normally performed by broth microdilution (BMD) according to Clinical and Laboratory Standards Institute (CLSI) or European Committee on Antimicrobial Susceptibility Testing (EUCAST) standards [46,47]. Interpretive breakpoints are available for ~8 (CLSI) and 14 (EUCAST) fungal species, including *C. auris* in the case of EUCAST [47,48]. Despite the limited number of species with defined breakpoints, BMD allows the generation of a minimum inhibitory concentration (MIC) for other species which has some clinical utility. BMD is laborious and specialized training is required [49], although commercial BMD systems can mitigate this somewhat [42]. Another limitation of BMD is trailing endpoints with triazoles, which represent a challenge to interpretation [42]. Other methods for susceptibility testing include disc diffusion, gradient strips and the commercial automated platform, VITEK2 [42,49]. To shorten the time to antifungal susceptibility results, a number of novel methods are also under investigation, including the use of MALDI-TOF MS, flow cytometry, calorimetry and microscopic growth assays [42]. PCR is a more rapid method but is generally limited to a small number of known resistance-associated mutations [10].

## Potential use cases for genomics in fAMR detection

Because WGS can identify fAMR-associated variants as well as infer transmission events, it carries the potential to aid public health authorities, infection control services and clinicians in (i) public health surveillance for emerging fAMR threats, (ii) local hospital outbreak management, (iii) culture-independent diagnosis of fAMR and (iv) potentially rapid detection of fAMR.

In particular, genomic surveillance can contribute to an understanding of the epidemiology of fAMR and its sources (for example, confirming an agricultural origin of a triazole resistant *A. fumigatus* clone) [50]. Genomics can enable tracking of antifungal-resistant clones in healthcare to inform infection control interventions (for instance, a multi-hospital fluconazole-resistant *C. parapsilosis* outbreak) [22]. Culture-independent diagnosis of fAMR is a key priority especially for immunocompromised patient cohorts to improve the sensitivity of culture-based detection [51] and finally the possibility of direct-from-specimen metagenomic sequencing to enable culture-independent fAMR detection [52], whilst noting the importance of patient selection and specimen quality to use this technology optimally [53].

Given the vulnerability of the patient cohort with invasive fungal infections, there is a critical need for diagnostics that are faster than culture-based methods. In addition to ascertaining susceptibility, there is a need for rapid identification of transmission of resistant pathogens (especially *C. auris*) in healthcare to limit the exposure of vulnerable patients [54]. Delays from batched WGS workflows may not be practical for clinical decision-making, but WGS nonetheless has the potential to serve as a rapid diagnostic, for instance, with the use of more flexible long-read sequencing technology [12].

## Current challenges in genomic fAMR detection

Relative to bacterial AMR, genomic correlates of antifungal resistance are complex and the evidence base for resistance mechanisms is less robust [55]. Fungi are eukaryotes, characterized by a membrane-bound nucleus and organelles, a nuclear genome segmented into multiple linear chromosomes and a mitochondrial genome. Fungal genomes are larger than bacterial genomes and genes may contain introns [56,58]. Some species are diploid, polyploid or demonstrate aneuploidies, complicating genome assembly [59] and variant calling due to heterozygosity, with variable impacts on antifungal resistance [60]. Diploid status also limits the utility of some variant calling software developed for haploid genomes [61]. Fungal genomes may be highly repetitive, contributing to genomic plasticity, notably in *C. albicans* [62], and the expression is influenced by chromatin modifications typical of eukaryotes. A further complexity is that the genetic code (translation schemes) varies between fungal genera: the nucleotide codon CTG is translated as leucine in most fungal species, but as serine in *C. albicans*, *C. auris* and related species (the so-called CTG clade) [63].

The cellular basis of antifungal resistance (such as drug target alteration, drug efflux and reduced drug activation) can be influenced by multiple forms of genetic alterations (Table 1). Genomic mechanisms impacting drug resistance include mutations, such as SNPs and small insertions and deletions (indels), tandem repeats (TRs), gene and chromosomal copy number variation and other forms of structural variation (SV) [55]. Each genomic mechanism requires a different form of analysis. However, genomic antifungal resistance tools and databases have focused largely on SNPs, omitting other important forms of genomic variation [16,27, 64].

Substantially, fewer fungal genomes and curated reference genomes are available in the public domain than for bacteria. Phenotypic data is often limited. For instance, NCBI RefSeq annotated genomes represent approximately 600 fungal species, but over 20,000 prokaryotic species [65]. Published genomic studies are often characterized by selection bias, with overrepresentation of phenotypically resistant isolates, complicating the interpretation of mutations. There are relatively few studies confirming the *in vitro* effect of mutations in site-directed mutagenesis models. In addition, surveillance programmes are more limited than for bacterial AMR. However, the WHO Global Antimicrobial Resistance and Use Surveillance System (GLASS) is preparing to integrate data on all major antifungal drug classes for *Candida* and related genera (GLASS-FUNGI) [23].

For public health laboratories performing genomic sequencing predominantly for bacterial pathogens, implementation of fungal genomic surveillance and development of validated tools will require familiarization with the complexities of eukaryotic genomics, drawing on expertise from the fungal and other eukaryotic research fields.

## Requirements and challenges for implementation of genomic detection of fAMR in public health

Translating genomic antifungal resistance detection from research into public health presents an additional challenge of demonstrating compliance with accreditation standards. Public health microbiology internationally is regulated by ISO standards including ISO 15189 [66]. As with any diagnostic assay, introduction of genomic antifungal resistance detection in public health microbiology requires a rigorous process of validation. Tools should be continuously maintained and should allow tracking of the whole analysis process [66]. Databases of resistance determinants must be transparently based on high-quality evidence, comprehensive, free from errors and continually curated. Version control and comprehensive test suites are essential for bioinformatic software and databases [66,67]. Further development of antifungal databases and tools should therefore reflect these requirements. For instance, databases should ideally include systematic grading of evidence and a controlled update process; and tools should include an audit trail function and preferably the ability to modularize processes to assist ongoing re-verification and re-validation.

Some of the challenges for database and tool developers are the requirement for ongoing database curation and software maintenance [68], which are tasks requiring a skilled team with sustainable funding [69]. Some of the current fAMR tools and databases have not been updated for several years, illustrating this challenge [70,71].

Challenges for laboratories considering implementation and ISO certification of genomic fAMR detection in public health might include low throughput of antifungal-resistant organisms, a paucity of validation datasets and unavailability of a validated analysis pipeline or bioinformatic expertise for all priority species (including diploid species with increasing fAMR, such as *C. parapsilosis*), software installation issues (as one illustration, the commonly used software environment manager Conda is not recommended for MycoSNP-NF by the authors because of the potential for dependency conflicts) [72] and subscription costs if using a cloud-based platform for analysis [73,74].

## Current databases and tools for genomic detection of fAMR detection

Publicly accessible databases that included a catalogue of specific genomic fAMR mechanisms with associated phenotypes were included in this review. Some tools and databases are developed specifically for use together, whereas others are standalone developments. The databases reviewed are in a range of formats, and databases developed for use with one tool or in isolation are not necessarily interoperable with other tools.

The databases included are (i) FungAMR [75], (ii) the Antifungal Resistance Database (AFRbase) [71], (iii) the Candida Genome Database (CGD) [76], (iv) the Fungal Resistance Database (FunResDB) [70], (v) the Pathogen–Host Interactions Database (PHI-base) [77], (vi) the TheiaEuk database [78], (vii) Pathogenwatch [79] and (viii) the FUNGAR database [52]. TheiaEuk is based on the previously publicly available database MARDy 1.0, which was developed by researchers at Imperial College London [78]. An updated version of MARDy (2.0) is currently in development [80].

Publicly available tools with an associated documentation (a publication, pre-print, repository or website) were included if they report fAMR mechanisms and/or inferred phenotypes from genomic data. The tools considered in this review are (i) Chromosome Query Targets (ChroQueTas) [75], (ii) the FunResDB search tool [70], (iii) MycoSNP-NF [79,81], (iv) Pathogenwatch AMR [79], (v) the TheiaEuk tool [73] and (vi) the FUNGAR tool [52].

Databases not included in this review included (i) ResFungi, a hidden Markov model-based protein database based on MARDy 1.0 [82]; (ii) FungiDB, a database of genome, sequence, protein and transcript data as well as an online tool for genome analysis [83]; (iii) MycoCosm, a database of fungal genomes and annotations [84]; and Saccharomyces Genome Database (SGD), a database of *Saccharomyces cerevisiae* sequences, genes and functions [85]. ResFungi was not included as its focus is the prediction of putative resistance-associated *genes* rather than the detection of resistance-associated variation (mutations) within those genes [82]. FungiDB, SGD and MycoCosm were not included, as, despite being comprehensive databases and useful tools for fungal genome analysis, they are not focussed on fAMR [85,86].

A comparison of databases and tools is presented in Tables2  3, respectively, including approximate quantitative data relating to database listings.

**Table 2.** Genomic fAMR databases reviewed

Database	Host	URL	Last update	Evidence type (human listings)	Metadata included	Human pathogen species (approx.)	Total entries (approx.)
**FungAMR** [75]	Université Laval, Canada	card.mcmaster.ca/fungamrhome	Apr 2025	Clinical Experimental	MIC Antifungal agent Host Study identifier Confidence score	98	35,792
**TheiaEuk database** [73]	Theiagen, USA	theiagen.github.io/public_health_bioinformatics/v2.2.1/workflows/genomic_characterization/theiaeuk/	April 2025	Clinical Experimental (Derived from MARDy 1.0)	Host Antifungal agent Study identifier	4	(~10*)
**The Fungal Resistance Database** (FunResDB) [70]	Leibnitz Institute for Natural Product Research and Infection Biology, Germany	elbe.hki-jena.de/FunResDb/	Apr 2017	Clinical	Categorical AST results Study identifier	1	109
**Candida Genome Database** [76]	Department of Genetics, Stanford University, USA	candidagenome.org	July 2025	Clinical Experimental	MIC Antifungal agent Study identifier	5	1,258
**Antifungal Resistance Database** (AFRbase) [71]	Department of Biophysics, University of Delhi, India	proteininformatics.org/mkumar/afrbase/	Nov 2023	Clinical Experimental	Host Antifungal agent Study identifier	35	3,691
**Pathogenwatch**	Centre for Genomic Pathogen Surveillance, UK	github.com/pathogenwatch/amr-libraries/blob/main/498019.toml	Mar 2023	Not stated	Antifungal agent Categorical AST	1	11
**The Pathogen-Host Interaction Database** (PHI-base) [77]	Rothamsted Research, UK	phi-base.org	March 2024	Clinical Experimental	Host Antifungal agent	4	5
**FUNGAR database** [52]	Henrique Antoniolli, Universidade Federal do Rio Grande do Sul, Porto Alegre, Brazil	github.com/resgen-br/fungar	Feb 2026	Clinical Experimental (derived from FungAMR)	Antifungal agent	10	904

NB: Total entries refers to fAMR-related entries with mutations listed, as listed in the publication or on the website, without exclusion of duplicates or erroneous entries, or if not stated, a count of publicly listed entries on the website or repository.

Human listings refers to listings of mutation/gene/species combinations where the mutation was identified in a pathogen isolated from a human patient. Human pathogen species refers to species known to have caused infections of humans. Genus-specific listings (e.g. in FUNGAR) are included in the species count.

*Ten *C. auris* entries listed in repository. For an additional three species, a total of eight genes listed, with total number of variants in these genes (as listed in MARDy 1.0 database) not stated.

AST, antimicrobial susceptibility testing.

**Table 3.** Bioinformatic tools used for genomic detection of fAMR included in this review

Tool	Host	URL	Last update	Input	Format	Output	Non-haploid variant calling	Alt. translation scheme	Code availability
**ChroQueTas** [75]	Université Laval, Quebec City, Canada	github.com/Landrylab/FungAMR	Aug 2024	Assemblies	CLI	VCF annotated with amino acid substitutions associated with resistance Confidence score for resistance-associated mutations	No (contigs only)	**Yes**	Yes
**The FunResDB search tool** (FunResDB) [70]	Leibnitz Institute for Natural Product Research and Infection Biology, Jena, Germany	elbe.hki-jena.de/FunResDb/	Sept 2019	SNPs ≤5000 bp amplicons	Web portal	Mutations detected Line list of published studies with categorical AST and concomitant mutations	No FASTA amplicon only	n/a	Yes
**MycoSNP-NF** [72]	Centres for Disease Control and Prevention, USA	github.com/CDCgov/mycosnp-nf	Dec 2024	Short reads	CLI	VCF – all VCF annotated with predicted amino acid substitutions (snpEff) (*C. auris* only)	**Yes**	No	Yes
**TheiaEuk 3.0.0** [61]	Theiagen, Highlands Ranch, USA	theiagen.github.io/public_health_bioinformatics/v2.2.1/workflows/genomic_characterization/theiaeuk/	Apr 2025	Short reads	Web portal CLI	VCF annotated with amino acid substitutions associated with resistance in MARDy 1.0	No	No	Yes
**Pathogenwatch AMR** [79]	Centre for Genomic Pathogen Surveillance, UK	github.com/pathogenwatch-oss/amr-search	Mar 2025	Assemblies Short reads *	Web portal CLI	Report with inferred antibiogram JSON output	No	No	Yes
**FUNGAR**	Henrique Antoniolli, Universidade Federal do Rio Grande do Sul, Porto Alegre, Brazil	github.com/resgen-br/fungar	Feb 2026	Short reads	CLI	CSV of mutations in each gene with associated antifungal drug class	No	Yes	Yes

* Short read inputs are assembled with SPAdes prior to SNP detection.

AST, Antimicrobial susceptibility testing; CLI, Command line interface; CSV, Comma-separated values; JSON, JavaScript object notation; SNPs, Single nucleotide polymorphisms; VCF, Variant call file.

The antifungal resistance databases were assessed on the following criteria: the number of entries and fungal species represented, including whether *C. auris* is included; the evidence types; associated metadata; the referencing; and any novel features. We assess features including the presence of obvious curation errors and missing data and the recency of updates. For the bioinformatic tools, we review the interface (command-line tool or web portal), the input and output file formats, the availability of source code, the tool’s capacity for more advanced functions including non-haploid variant calling and capability for alternative genetic codes and any novel features. We consider whether the tool has been evaluated in a peer-reviewed publication. Finally, we assess the accessibility of the tool and the clarity and utility of the output for the end-user. We consider specific strengths and limitations of each database and tool.

### FungAMR and ChroQueTas

FungAMR is a large database of antifungal resistance determinants released in 2024, developed at the Landry Lab at l’Université de Laval, Quebec City, Canada, in collaboration with a group of experts at institutions in Canada, the Netherlands and Spain. ChroQueTas is the command-line tool developed by this group for use in conjunction with FungAMR [75].

FungAMR contains ~35,792 entries, including those relevant to 98 human pathogens, including *C. auris*. It is manually curated from ~501 papers. Evidence types range from human clinical studies to experimental evolution and site-directed mutagenesis studies. FungAMR includes both mutational (amino acid mutations) and non-mutational (such as deletions, aneuploidies, overexpression and gene disruption) mechanisms. Metadata included are the medication affected; the strain identifier; the source of the listing and the MIC, fold change in susceptibility or other measure of phenotypic antifungal susceptibility where available. The last update appears to have been in April 2025 [87].

A novel feature is the introduction of the confidence scoring (evidence grading) system. This is an arbitrary scale from 1 to 8, with 1 the strongest positive confidence score (mutation created in susceptible pathogen, resulting in resistance) to 8 (mutation found in a natural isolate). Each entry has a confidence score for resistance and susceptibility; for instance, if a mutation has been confirmed to be resistance-causing *in vitro*, it will have a score of +1 for resistance evidence and −1 for sensitivity evidence.

The strengths of FungAMR are its comprehensiveness, the completeness of the metadata and referencing, a minimal use of gene synonyms and a low frequency of obvious curation errors for human pathogen listings (such as incorrect orthologue names or incorrect gene/drug class associations). Although not directly relevant to human mycology, the ‘One Health’ approach of including plant and animal pathogens is forward-looking. A limitation is the representation of non-mutational mechanisms which are non-specific (e.g. ‘aneuploidy’). Whilst an important addition to the fungal database field, the confidence scoring system does not provide any differentiation between clinical studies (for instance, clinical studies vary in sample size and the extent of representation of susceptible controls), and the system for summarizing entries only takes the highest and lowest representatives, which does not capture the distribution of confidence scores. Confidence scores should not be taken as directly implying the probability of a resistant phenotype for clinical or public health use.

ChroQueTas is a shell script tool for screening fungal genomes for coding sequence mutations (SNPs and insertions/deletions) included in FungAMR [75,88]. The input type is a FASTA assembly (contigs) file. The output is a series of gene-specific tab-separated value files of mutations by amino acid position and the FungAMR confidence score, and a summary file of mutations is identified. ChroQueTas incorporates alternative genetic codes in the species selection. An evaluation of ChroQueTas using 46 *C*. *albicans* and 144 *Zymoseptoria tritici* genomes has been reported in a peer-reviewed publication, although formal validation and comparison to other tools are absent to date [75].

The strengths of ChroQueTas are (i) the ability to deal with multi-exon genes, which is important for some fungal species, (ii) ability to report mutations with FungAMR confidence scores and (iii) selection of a genetic code (codon translation table), allowing for the alternative translation scheme of the CTG clade [75].

Limitations of ChroQueTas are (i) need for an assembled genome, which limits its ability to deal with minor populations and heterozygosity and potentially introduces variability due to users employing various assembly approaches; (ii) analysis of coding sequences only, excluding mutations in non-coding regions such as promoters; and (iii) limitation to SNP and indel detection (for instance, TRs, important in resistance detection for *A. fumigatus*, are not yet able to be detected).

The FungAMR database is available on the CARD database website (card.mcmaster.ca/fungamrhome) as of December 2024 [75,89]. The source code for ChroQueTas is publicly available on GitHub [88]. ChroQueTas can be installed via Conda [90] or via the source code.

### AFRbase

AFRbase [71] is a web browser-based database of amino acid mutations released in 2023 by a team at the Department of Biophysics, University of Delhi South Campus, India. The web portal includes a blast function and protein structure visualization tool. Information sources reported include manual curation and text mining of literature retrievable through PubMed, although the specific text mining algorithm used is not described.

AFRbase contains 3,691 entries relevant to humans, animals and plants, covering 35 human-pathogenic species. Sources of mutations are from clinical and *in vitro* studies. Missense and nonsense mutations are included. There are ~12 entries for mutational combinations. The last update was September 2023 [91].

The key strength of AFRbase is its comprehensiveness and inclusion of plant and animal pathogens. There are however several limitations to note. Missing core data is frequent (species name missing *n*=265, gene name missing *n*=787 and mutation missing *n*=3). Missing metadata is also frequent (host type missing *n*=3,374). In the absence of a controlled ontology, there is a frequent use of gene synonyms, one consequence of which is duplicate listings (for instance, duplication of entries for *FKS1* and *GSC1*). Association errors (wrong species reported for a published mutation/gene combination; wrong gene reported for a mutation/species combination) are common. Incorrect orthologue names occur (e.g. *ERG11* for *Aspergillus flavus*). Importantly, there are numerous erroneous mutation entries which appear to result from imputation by the text mining algorithm of an amino acid mutation (in the form ‘letter-number-letter’) where the text element was in fact a company name, an alphanumeric postcode element, a medical dosing abbreviation or similar. Of note, AFRbase is in use by the genomic AMR surveillance platform Solu [92], so improvement to its curation process is a priority.

AFRbase is available from proteininformatics.org/cgi-bin/afrbase/.

### Candida Genome Database

The CGD [93] is a large web portal-based database of genomes, genes and nucleotide and protein sequences hosted by Stanford University, USA, since 2004 and funded by the National Institute of Dental and Craniofacial Research and the US National Institutes of Health [76]. The web portal includes a genome browser and blast function [93].

CGD covers five yeast species: *C. albicans*, *N. glabratus*, *C. parapsilosis*, *Candida dubliniensis* and *C. auris*. CGD contains ~1,258 records relating to fAMR (i.e. after excluding records in the categories ‘resistance to chemical: absent’ or ‘decreased’), including 306 with specified mutations that relate to antifungal classes used or being trialled in humans, including mutations (including amino acid substitutions, indels, truncations and gene deletions) and gene overexpression or deletion. Listed mutations are derived from both clinical isolates and *in vitro* evolution. CGD is based on manual literature-based curation [93]. The metadata includes the medication affected, phenotypic antifungal susceptibility (MIC) and strain and experimental details.

Strengths of CGD as an fAMR database include its recency of curation (includes publications from 2025) and consistent referencing of entries. Limitations include incompleteness of metadata for fAMR entries: for instance, MIC data is present in only ~141/306 human fAMR mutational records. Non-mutational mechanisms are presented non-specifically (e.g. ‘overexpression’). MICs are presented in non-standardized free-text formats. Some curation issues are present, mostly relating to the lack of a controlled vocabulary: for instance, either ‘azole’ and ‘triazoles’ is used, both ‘flucytosine’ and ‘5-fluorocytosine’ are used, and the gene synonym *GSC1* is used in place of the more widely accepted *FKS1* for *C. albicans*.

The CGD is available from candidagenome.org.

### The FunResDB

FunResDB is a single-gene, single-pathogen database of published associations between *CYP51A* genotypes and triazole resistance phenotypes in *A. fumigatus* released in 2017 and hosted by the German National Reference Centre for Invasive Fungal Infections, Jena, Germany [70]. FunResDB claims to be the first publicly available web tool for sequence-based antifungal susceptibility screening [70]. FunResDB is accompanied by a web portal tool for resistance-associated mutation screening (referred to here as the FunResDB tool).

FunResDB contains ~109 listings, each with an associated phenotype (susceptible, intermediate or resistant to one of four triazole antifungals) and a publication reference (complete in all cases). The database is manually curated from PubMed (NBCI, National Library of Medicine, USA). FunResDB is able to deal with the two-exon nature of *CYP51A* in *A. fumigatus* by including spliced and unspliced FASTA references. It also has the capacity for promoter TR detection. FunResDB was last updated in April 2017. The FunResDB tool has been evaluated in a peer-reviewed publication with a relatively small in silico dataset, demonstrating correct detection in a collection of 18 resistant isolates [70].

The strengths of FunResDB as a database include its comprehensiveness for fAMR determinants in the studied gene and the completeness of the phenotypic data and referencing. The main limitation is the recency of curation.

The FunResDB tool accepts an input of short nucleotide (up to 5 kb) or protein sequences or individual mutations. The output is a web table listing all occurrences of the mutation in the included publications with the associated phenotypes. Each phenotype (combination of categorical antifungal susceptibility findings) is represented as a separate line list (for instance, for the queried single mutation G448S, four listings are provided for resistance and one for susceptibility to itraconazole).

The main strengths of the FunResDB tool are its web browser interface which is suitable for non-bioinformaticians; the ability to report mutational combinations and promoter TR variation and properly handle the presence of an intron are significant advantages for detecting triazole resistance in *A. fumigatus*. The simple and easily interpreted output is another strength.

The main limitation of the FunResDB tool is the maximum sequence length accepted being only 5,000 bp; whilst suited to Sanger sequencing, it is not compatible with whole-genome sequence reads or assemblies. It is also unable to deal with heterozygosity and non-mutational mechanisms [70].

FunResDB and its associated tool are available from funresdb.hki-jena.de and source code from github.com/mweberr/FunResDb.

### The Pathogen–Host Interaction Database

PHI-base is a database of pathogen–host interactions initiated in 2005, presented as a searchable web portal and hosted at Rothamsted Research, UK [94].

PHI-base is a manually curated database of genes and mutations in fungal, bacterial and parasitic pathogens with experimental evidence. PHI-base predominantly contains pathogens of plants but includes some affecting human, animal and insect hosts [77]. The last update to PHI-base was in February 2026. Metadata included are the host species; the disease state (e.g. dermatophytosis); strain details; gene, protein and annotation accessions; and the phenotype. Each record includes a reference (including digital object identifier) and the publication database (e.g. PubMed).

PHI-base contains a small number of fAMR mechanisms although the database curators plan to increase coverage of antifungal resistance determinants in the future [77]. PHI-base includes two *ERG1* (squalene epoxidase) mutations conferring terbinafine resistance in *Trichophyton rubrum*, three listings for *ERG1/ERGA* variants in *Aspergillus* spp. (two mutational, one SV) and four concomitant *PDR1* SNPs in association with triazole resistance in *N. glabratus* [95].

Strengths of PHI-base include its ongoing curation and links to other databases. From a fAMR perspective, the key limitation of PHI-base is its limited coverage of fAMR mechanisms. The categories used to list fAMR mechanisms [typically ‘increased virulence (hypervirulence)’ or ‘chemistry target: resistance to chemical’] could be modified for clarity. Mutations are typically listed as a string under ‘Allele’ (e.g. ‘G1099D; P927S; L344S; G346D’).

PHI-base is available from phi-base.org.

### TheiaEuk

The TheiaEuk pipeline [73], developed by Theiagen Consulting LLC and first released in 2023, is an automated workflow for fungal variant calling with the capacity to report resistance mutations. TheiaEuk can be accessed on a command line or web interface (via the Terra.bio cloud platform). TheiaEuk is accompanied by a small fAMR database which the authors state is derived from the MARDy 1.0 database [96].

The TheiaEuk database comprises 11 species–gene combinations, encompassing *C. auris* (six mutations), *C. albicans*, *A. fumigatus* and *Cryptococcus neoformans*. Only the *C. auris* mutations are published on the website: three *ERG11* mutations relevant to triazoles, two *FKS1* affecting echinocandins and one flucytosine resistance mutation in *FUR1*. Metadata are host type, gene locus, medication affected and references.

The TheiaEuk tool takes an Illumina FASTQ read input, performs data quality control (QC) and species identification and haploid reference-based variant calling and searches the database for fAMR mutations. Outputs include read and assembly quality metrics, a summary of the variants detected. TheiaEuk has been evaluated in a peer-reviewed publication [73].

A strength of the TheiaEuk pipeline is its ability to report fAMR mutations directly from raw reads, including performing data QC and *C. auris* clade assignment, increasing its accessibility to non-bioinformaticians. The performance of TheiaEuk has been evaluated in a peer-reviewed publication, demonstrating a high level of accuracy in resistance mutation detection in three genes and clade typing for *C. auris* [73]. Limitations of TheiaEuk include the small size of the database and its limitation to haploid variant calling only, despite accepting raw reads and the listing of diploid species in the database [61]. For use in its web portal format, local regulations might restrict uploading of data to a third party.

The command line version of TheiaEuk can be installed via Docker [97]. The database and source code are available at theiagen.github.io/public_health_bioinformatics/v2.2.1/workflows/genomic_characterization/theiaeuk. The web portal version is hosted on Terra.bio, which may require a subscription for organizations [98].

### MycoSNP-NF and GATK

MycoSNP-NF is a command-line workflow developed by the US Centers for Disease Control and Prevention in 2022, based on the Genome Analysis Toolkit version 4 (GATK 4) variant caller [99] and using Nextflow for automation and logging. MycoSNP-NF has also been released by Theiagen in a Workflow Description Language (WDL) format [100] for use from the Terra.bio platform [74].

MycoSNP-NF accepts Illumina FASTQ files reads from haploid or diploid organisms and performs haplotype-aware variant calling. The main output is a variant call file (VCF). For *C. auris* only*,* MycoSNP-NF also reports amino acid substitutions in *FKS1* using snpEff [72,81]. MycoSNP has been evaluated for *C. auris* variant calling, showing comparable performance to other variant calling pipelines including TheiaEuk for *C. auris* [101].

Strengths of MycoSNP-NF are the ability to perform all tasks from read input to variant calling, the capacity for workflow auditing enabled by Nextflow and reporting of amino acid substitutions in *FKS1* hotspots for *C. auris*. Being based on GATK, MycoSNP-NF has the capacity for diploid variant calling, which is relevant to important fungal pathogens including *Candida albicans*. GATK has been extensively used in fungal genomics and has been shown to perform well in a comparison with other pipelines for *C. auris* [102].

Limitations of MycoSNP-NF include an inability to detect TRs and copy number variants and the lack of fAMR variant annotation for species other than *C. auris*.

The source code for MycoSNP-NF is available from the GitHub repository (github.com/CDCgov/mycosnp-nf). MycoSNP-NF can be deployed via Docker or Apptainer containers, but significantly, Conda is not recommended by the authors because of the potential for dependency conflicts.

### Pathogenwatch AMRsearch

Pathogenwatch AMRsearch (PW-AMRsearch) is a pipeline developed by the Centre for Genomic Pathogen Surveillance (Oxford, UK) for pathogen identification, typing and detection of resistance mechanisms and virulence factor genes [103]. The first application to a fungal pathogen (*C. auris*) was in 2023 [104].

PW-AMRsearch can be used via a graphical user interface (GUI) or on the command line. The input types are assemblies, or FASTQ short reads which are then assembled within the pipeline [79]. The input genome size is limited to 20 Mb. PW-AMRsearch enables blast-based mutational resistance detection in *C. auris* only. The output of PW-AMRsearch is a genome report including an inferred phenotype (‘resistant’ for an associated antifungal drug where a resistance-associated mutation is identified) [79]. PW-AMRsearch correctly identified all occurrences of three mutations in *ERG11* (K143R, Y132F and F126L) in *C. auris* in the evaluation by Li and colleagues [102].

PW-AMRsearch is accompanied by a limited *C. auris* database of 11 SNPs across five genes (*ERG11, CDR1, FKS1, FUR1* and *FCY1*) relevant to three drug classes, the triazoles, the echinocandins and flucytosine [102,105].

A key strength of PW-AMRsearch is the generation of an inferred antibiogram. Limitations include its limitation to a small number *C. auris* mutations; the use of an assembly input, which does not allow detection of heterozygous variants or minor alleles; and an apparent lack of internal quality control within the pipeline (the authors recommend users check their assembly quality if unusual results are encountered) [106], potentially reducing its accessibility to non-bioinformaticians. As with some other fAMR pipelines, uploading of data to the web portal may be restricted by local regulations.

The Pathogenwatch source code is available from github.com/pathogenwatch-oss/amr-search. PW-AMRsearch can be downloaded via Docker or via the source code.

### FUNGAR

FUNGAR is a recently released (2026) command-line tool developed by the Universidade de Rio Grande do Sul, Brazil. Its stated aim is detecting fAMR mutations from metagenomic sequence reads [52].

FUNGAR accepts a paired- or single-end FASTQ short read input, performs an alignment with DIAMOND, queries a database of amino acid mutations and outputs a CSV with a line listing for each antifungal drug associated with the mutation. The pipeline allows specification of the genetic code.

FUNGAR’s database contains ~904 amino acid mutations listed with the affected antifungal drug class. The database contains seven sets of species-specific mutations (*A. fumigatus*, *A. flavus*, *Aspergillus niger*, *Aspergillus terreus*, *C. neoformans*, *Histoplasma capsulatum* and *Pneumocystis jirovecii*) and three sets of genus-specific mutations (*Aspergillus, Cryptococcus* and *Candida*). The authors state their database is derived from FungAMR [52]. A small validation study is described in a preprint, demonstrating correct fAMR mutation detection in a dataset of 10 isolates and 11 metagenomes [52].

A strength of FUNGAR is the direct output of resistance-associated mutations from a read input. Use of reads directly avoids potential errors from assembly which cannot be controlled for in an assembly-input tool. Limitations in its current form include the lack of confidence scores, phenotypic susceptibility data and references for fAMR records. Thus far a limited number of species are included (for instance, *C. albicans* but not *C. auris* or *N. glabratus*).

FUNGAR’s source code can be obtained from github.com/resgen-br/fungar.

## Future directions

Whilst significant advances have recently been made into genomic determination of antifungal resistance, the field is still in an early stage of development relative to the bacterial AMR field. Database comprehensiveness and quality has substantially improved with the release of FungAMR in 2024, although there are some limitations including the representation of SVs. For clinical and public health microbiology, there is a lack of end-to-end workflows that have been validated to ISO standards for genomic antifungal resistance reporting. As a result, developing databases and tools for accurate and reproducible AMR detection in fungi is an important research priority.

**Fig. 2.**
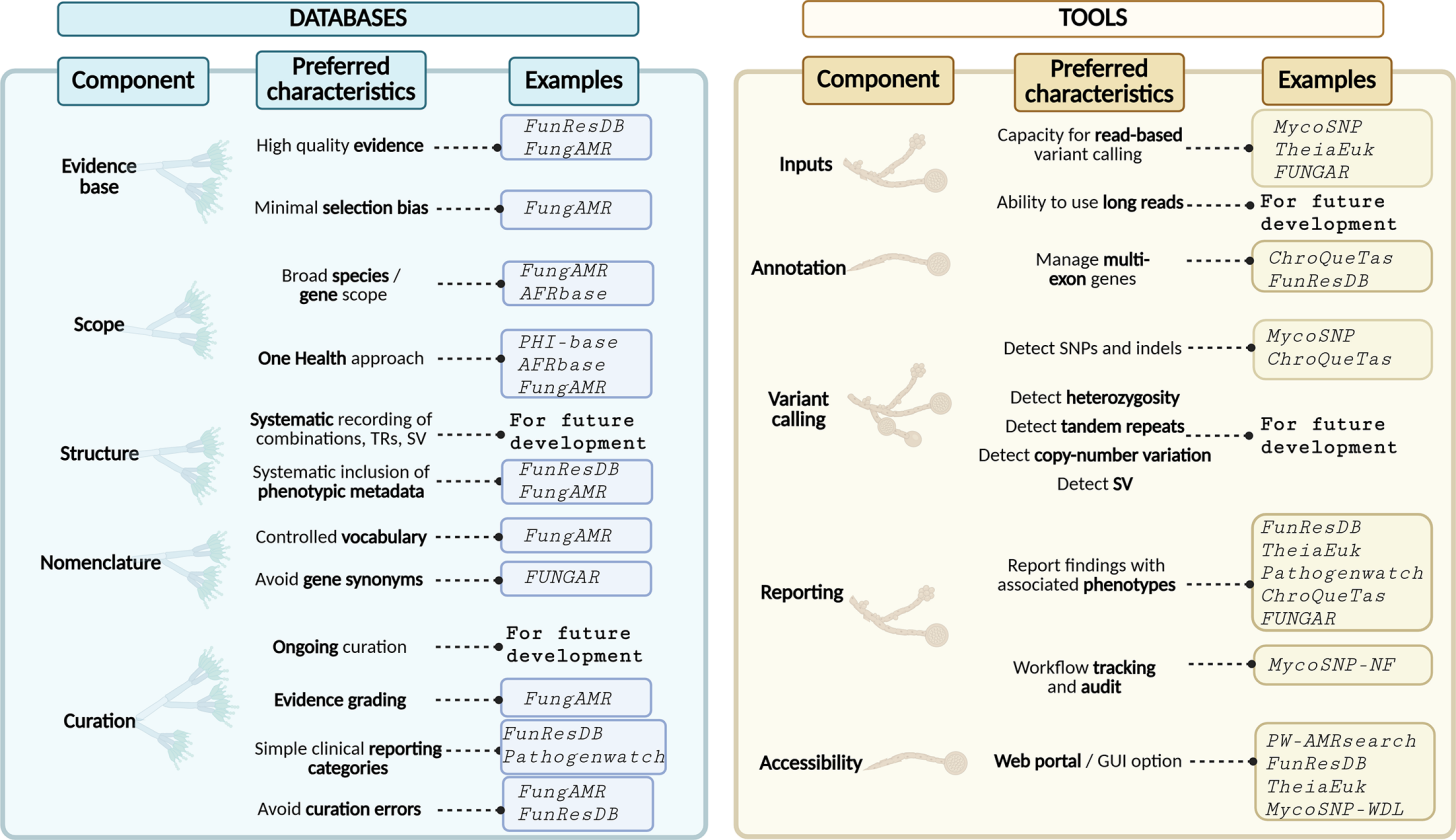
Preferred characteristics of antifungal resistance genomic databases and tools with current examples.Created in BioRender. Gador-whyte, A. (2026) https://BioRender.com/f3hm130

Fig. 2 summarizes our assessment of preferred product characteristics for the further development of antifungal genomic databases and tools, considering regulatory requirements, the unique genomic resistance mechanisms of fungal pathogens and the practical requirements of a clinical or public health laboratory.

### Advancing evidence for antifungal resistance mechanisms

Arguably, the largest limitation to antifungal databases is the limited evidence base supporting phenotypic–genomic associations. Building the evidence base for mutational resistance determinants will require a more systematic approach to assessing the impact of single and combined mutations on phenotypes, with selection bias systematically addressed so that resistant isolates are not over-represented in studies (which limits the downstream specificity of identification of these mutations in clinical isolates). Use of a common controlled vocabulary (ontology) would also facilitate the sharing of data to improve the robustness of the evidence base [55].

Additionally, a greater use of both *in silico* modelling (such as *in silico* evolution and protein-modelling approaches) and traditional *S. cerevisiae* mutational expression studies would greatly improve the balance of evidence for the listed genomic mechanisms. CRISPR-Cas9 also represents an opportunity to expedite experimental validation of nonsynonymous mutations in fAMR genes [8].

### Standardization and validation for public health use

The bioinformatic tools reviewed are heterogeneous in their input types (short reads, assemblies and amplicons), variant calling software and reporting approaches used. The outputs also vary in the level of referencing and supporting evidence claimed. Databases are also in a variety of formats. Establishing minimum standards for fAMR databases and tools would be a step towards greater quality, standardization and harmonization. Minimum standards for databases to be used in public health microbiology should include source data, complete categorical (susceptible/resistant) or numerical (MIC) phenotypic AFST results and an explicit process for curation and addition of future entries. Curation should avoid unsupervised data mining techniques given the substantial scope for errors in assigning genomic variants to species and phenotypes. Ideally, databases should be in a systematic format to maximize interoperability.

A preferable characteristic of tools is the processing of FASTQ reads, with assembly performed within the pipeline if necessary, to minimize heterogeneity from the use of various assemblers by software users. Tools should ideally also accept a haploid or diploid VCF but should be able to perform the amino acid substitution annotation to systematize this step. Workflow auditing and the ability to perform each analysis step in a modular fashion can aid the process of verification by the implementing laboratory as required under ISO standards [66].

Implementing laboratories need to assess the performance of bioinformatic tools for the required organisms and input types, in terms of the sensitivity, specificity, limit of detection (such as minimum average sequencing depth for reliable results), reproducibility and other metrics [67] and data QC thresholds should be specified [66]. Although some fAMR pipelines include reporting of inferred drug resistance phenotypes from genomic data [79], rigorous validation is required for implementation in clinical and public health microbiology [67], especially if genomic AST reporting is ultimately used for clinical decision-making in the future.

### Flexible analysis workflows allowing for varying requirements between pathogen species

Ideally, a validated bioinformatic pipeline would be capable of modifying analysis based on the input species, to maximize the appropriateness of the analysis and the utility to the end-user. For instance, for *C. auris,* haploid variant calling is generally considered adequate and clade determination is important for public health purposes. Conversely, for many *Candida* species, diploid variant calling is required; diploid sequence typing may be of value to the end-user where a scheme exists, for instance, for *C. albicans* [107]. The fAMR genes of interest also vary between species (for example, *ERG11* and *TAC1B* for *C. auris; PDR1* for *N. glabratus*) [108,109]; hence, a tool should ideally have the capability to select genes of interest based on the species input.

### Inclusion of resistance mechanisms for novel agents

Several novel antifungal drugs representing new antifungal classes are in varying stages of development: fosmanogepix (oxazole or Gwt1 inhibitor class), olorofim (orotomide), ibrexafungerp (triterpenoid), otesaconazole (tetrazole) and the GCN5 and histone acetylation inhibitor CPTH2 [110,111]. In addition, there is a new long-acting member of the echinocandins (rezafungin) which is likely to be used increasingly for antifungal prophylaxis in immunocompromised patients [112]. The development of these novel agents warrants close surveillance for resistance. It is critical that research is conducted into mechanisms of resistance, with validation of genomic findings and their inclusion in genomic antifungal resistance databases as clinical resistance emerges.

### One Health approach

FungAMR has taken an encouraging step of including many listings of fAMR determinants in agricultural pathogens (1,041 listings). Although many fungal pathogens of plants are not pathogenic to humans, there is an overlap (for instance, *Fusarium solani* as an aggressive pathogen of both humans and crops) [113]. Of particular concern is the impact on human health of antifungal resistance emerging in agriculture (most importantly in *A. fumigatus*) through the use of fungicide classes with human health applications. For instance, the licencing of the orotomide ipflufenoquin for agricultural use in the USA and Australia might compromise the novel agent olorofim [112,114]. Understanding and validating genomic mechanisms of resistance in agricultural and environmental pathogens might contribute to surveillance for and rapid detection of resistance where these or related species also pose a human health threat.

### Further developing confidence scoring

FungAMR represents, to our knowledge, the first systematic approach in a fungal genomic database to grading the level of evidence for or against a mutation as a resistance determinant (confidence scoring). Although this is a useful way of expressing the evidence level, this could be further developed as an interpretive tool. Firstly, simple reporting categories (for instance, ‘associated with resistance’, ‘uncertain significance’ and ‘not associated with resistance’), modelled on the WHO Tuberculosis Mutation Catalogue [115], would aid in reporting of inferred phenotypes for clinical end users. Secondly, the mechanism for summarizing confidence scores across cited studies could be further developed to take account of the evidence provided by recurrent appearance of a mutation in phenotypically resistant clinical isolates. The current methodology reports the highest and lowest confidence scores, but the categories 8 and −8 (appearance in nature without functional confirmation) could be subdivided to differentiate frequent independent occurrences in clinical isolates from, for instance, a mutation reported once only. Where known resistance ‘hotspots’ occur in fAMR genes, for instance, in *FKS1* in many yeasts [116] and *PDR1* in *N. glabratus* [117], the location of inferred amino acid substitutions could be incorporated as supporting evidence for a mutation as a resistance determinant.

### Integrating SV and epigenetics into resistance prediction

SV such as segmental aneuploidies and chromosomal deletions can play a significant role in fAMR [62]. For example, the i5L isochromosome in *C. albicans* results in an *ERG11* copy number increase [39]. Triazole-resistant, respiratory-deficient petite variants in *N. glabratus* and other yeasts can result from mitochondrial genome deletions [118]. Copy number variation can be predicted from short-read sequencing using read mapping approaches [119]. However, defining SVs to understand their impact on gene dosage and resistance is a current challenge for fungal bioinformatics [64]. Long-read sequencing, with its ability to enable phased non-haploid chromosome assemblies and to resolve large chromosome-level changes and long repeat regions (albeit not whole-genome duplication) [120] is likely to contribute substantially to this field.

A further benefit of some long-read sequencing techniques (Oxford Nanopore and PacBio SMRT Sequencing) is the ability to detect epigenetic changes such as DNA methylation through sequencing of the native molecule [121,122]. Epigenetic modifications are important mechanisms behind altered expression of drug targets and efflux pumps as well as other stress responses, allowing persistence in the presence of antifungal drugs [123,124]. Detection of epigenetic changes might become an important component within a multimodal analysis pipeline for understanding complex resistance mechanisms in the future.

### Improving accessibility

Given access to bioinformatic expertise is often limited and bioinformatic workforce and training challenges exist, in both low-income [125] and high-income settings [126,127], implementation of genomic antifungal resistance detection in public health laboratories will be aided by improvements to (i) accessibility of user interfaces and reports, (ii) data capacity of web browser-based tools and (iii) affordability of subscriptions where third-party hosting platforms are used. FunResDB, Pathogenwatch and TheiaEuk are examples of GUIs which may be more easily used by the non-bioinformatician, although FunResDB and the Pathogenwatch web portal format have strict input data size limits. On the other hand, the graphical interface options for TheiaEuk and MycoSNP-WDL may require a subscription to the hosting cloud platform, which may represent a barrier to low-resource settings.

## Limitations

This review limited itself to publicly available databases with associated documentation. As such, this review did not directly consider databases and tools in development or highly curated databases that are not currently publicly available, such as MARDy 1.0. Whilst currently under development, MARDy 2.0 is set to contain data from 413 publications, with 92 fungal species, 212 genes associated with resistance and 187 antifungal drugs represented. In comparison, MARDy 1.0 only contained data from 60 publications, with 22 fungal species, 25 genes and 25 antifungal drugs. It was also beyond this scope to review non-genomic molecular surveillance methods, such as PCR- and MALDI-TOF MS, comprehensively.

## Conclusion

Antifungal resistance is increasing globally. WGS is a powerful tool increasingly used for multiple purposes, including public health surveillance, hospital outbreak control and resistance detection. In these circumstances, the ability to detect resistance in invasive fungal pathogens reproducibly from genomic data will become increasingly useful. Whilst recent developments in databases have represented a substantial improvement, there is a need to build on the evidence base supporting phenotypic–genomic correlation. The field of bioinformatic tools is also advancing significantly, but further development is required to capture the complexities of antifungal resistance and integrate these tools into validated public health workflows.

## Glossary

**Table:** 

Term	Definition
Aneuploidy	Having an atypical number of chromosomes
Bioinformatics	A scientific discipline dealing with the computational analysis of biological data, drawing on knowledge from biology, statistics and computer science
Bp	Base pairs: number of individual nucleotides (adenine, guanine, cytosine, thymine) in a DNA sequence of interest; kilobases (kb): thousands of base pairs; megabases (Mb): millions of base pairs
Curation	Practice of systematic assessment of data quality for inclusion in a database of genomic resistance determinants
Diploid	Having two sets of chromosomes
Eukaryote	Organism having eukaryotic cell type, that is, containing membrane-bound nucleus and organelles (including all plant, fungal, protozoal and animal cells)
Gene dosage	The number of copies of a given gene (which may influence the final level of expression of a given gene product such as an enzyme)
Haploid	Having one set of chromosomes
*In silico*	Analysis using computer modelling
Indel	Abbreviation of ‘insertion or deletion’ Type of genetic change (mutation) occurring during DNA replication in which the DNA copy contains a short string of additional nucleotides (insertion), or a short deletion of nucleotides; generally refers to modifications of less than 50 base pairs
Isochromosome	A form of chromosomal structural variation in which a novel chromosome is formed from two copies of the long or short arm of a native chromosome
Laboratory validation	A rigorous process of systematically assessing the fitness for purpose of a newly developed laboratory assay or whole workflow against a given standard, such as ISO 15189; in microbial genomics, includes testing an adequate sample of representative specimen and micro-organism types; typically includes measuring the sensitivity, specificity, accuracy, precision, reproducibility and limit of detection
Ontology	A systematic, hierarchical and controlled vocabulary (for instance, used to standardize recording of genes, organisms and drug classes in some genomic databases)
Recombination	Exchange or copying of genetic material between chromosomes, either within or between cells
SNP	Type of genetic change (mutation) occurring during DNA replication in which the DNA copy contains a different nucleotide at a given position along the nucleotide sequence
Tandem repeat	A series of repetitive duplications occurring in the genome; tandem repeats adjacent to a gene’s coding sequence may result in changes of expression of the gene

## Supplementary material

10.1099/mgen.0.001710 Supplementary Material 1.

## Abbreviations

AFRbase: Antifungal Resistance Database
AFST: antifungal susceptibility testing
AMR: antimicrobial resistance
BMD: broth microdilution
CGD: Candida Genome Database
ChroQueTas: Chromosome Query Targets
CLSI: Clinical and Laboratory Standards Institute
EUCAST: European Committee on Antimicrobial Susceptibility Testing
fAMR: fungal antimicrobial resistance
FunResDB: Fungal Resistance Database
GATK: Genome Analysis Toolkit
GLASS: Global Antimicrobial Resistance and Use Surveillance System
GUI: graphical user interface
MIC: minimum inhibitory concentration
PHI-base: Pathogen–Host Interaction Database
PW-AMRsearch: Pathogenwatch AMRsearch
SGD: Saccharomyces Genome Database
SV: Structural variation/variants
TR: tandem repeat
VCF: variant call file
WDL: Workflow Description Language
WGS: whole-genome sequencing
WHO: World Health Organization
