# Supplementary material for: Towards accurate genomic detection of fungal antimicrobial resistance: progress in fungal resistance databases and bioinformatic tools
Source: Microb Genom. 2026 May 20;12(5):001710. doi: 10.1099/mgen.0.001710 (PMC13189648; doi:10.1099/mgen.0.001710)
Supplement: Supplementary Material 1. [file mgen-12-01710-s001.pdf]

SUPPLEMENTARY MATERIAL

1. Search strategy

The PubMed database was searched using the following search terms:

("drug resistance, fungal"[MeSH Major Topic]) AND ("databases as topic"[MeSH Major Topic])

(("databases as topic"[MeSH Major Topic]) AND ("genomics"[MeSH Major Topic])) AND ("fungi"[MeSH Major Topic])

("databases, factual"[MeSH Terms]) AND ("antifungal agents"[MeSH Terms])

(("workflow"[MeSH Terms]) AND ("genomics"[MeSH Terms])) AND ("genome")

((drug resistance, fungal[MeSH Terms]) AND ("candida"[MeSH Terms])) AND ("genomics"[MeSH Terms])

("databases as topic"[MeSH Terms]) AND ("antifungal agents"[MeSH Terms])

((("whole genome sequencing"[MeSH Terms]) AND (antifungal agents[MeSH Terms])) AND ("workflow"[MeSH Terms])

(("drug resistance, fungal"[MeSH Terms]) AND ("candida"[MeSH Terms])) AND ("genome, fungal"[MeSH Terms])

2. Publication inclusion criteria

The following criteria were used to select publications with relevance to the review

1. Publications referring to databases that include records of genomic variants in potential fungal antimicrobial resistance (fAMR) genes in fungi (excluding databases of genes only without specific variants)
2. Publications referring to tools specifically for use in detecting potential antimicrobial resistance-associated variants in fungal pathogens.

3. Database and tool inclusion criteria

The final list of databases was established by applying the following inclusion criteria:

1. publicly accessible
2. include a catalogue of specific genomic fAMR mechanisms / variants with phenotypes

The final list of tools was established by applying the following inclusion criteria:

1. publicly accessible
2. associated documentation (publication, pre-print, repository or website; not abstract alone)
3. tool reports fAMR mechanisms and/or inferred phenotypes from genomic data

4. Search results and publication, database and tool selection

The initial search yielded 549 records. 63 publications met initial inclusion criteria. Multiple publications might reference a given tool or database. Additionally, some publications described variant calling software (including variant callers used within fungal workflows) but not a fungal-specific workflow per se.

After de-duplication and inclusion of additional databases and tools identified by the authors through other sources including academic networks, a final 8 databases and 6 tools were included in the review.
